# Expression of adiponectin and its receptors in type 1 diabetes mellitus in human and mouse retinas

**Published:** 2013-08-04

**Authors:** Tao Lin, Yiguo Qiu, Yu Liu, Rajiv Mohan, Qiuhong Li, Bo Lei

**Affiliations:** 1 Department of Ophthalmology, the First Affiliated Hospital of Chongqing Medical University, Chongqing Key Laboratory of Ophthalmology, Chongqing Eye Institute, Chongqing, China; 2 Mason Eye Institute, School of Medicine, University of Missouri-Columbia, 1 Hospital Dr., Columbia MO; 3 Department of Ophthalmology, University of Florida, Gainesville, FL

## Abstract

**Purpose:**

Recent studies have suggested that adiponectin (APN) is associated with several retinal diseases. We studied the expression of APN and its receptors (AdipoRs) in the human retina and in a mouse model of type 1 diabetes mellitus (T1DM).

**Methods:**

Human eyeball specimens were obtained from the Chongqing Eye Bank. eNOS-knockout (*eNOS^−/−^*) mice were randomly divided into a T1DM group and a control group. The T1DM model was induced with an intraperitoneal injection of streptozotocin. To locate the AdipoRs in the retina, immunofluorescence was performed. Total APN protein and RNA were extracted from the neural retina and the retinal pigment epithelium (RPE)-choroid complex, and the APN protein was detected with enzyme-linked immunosorbent assay (ELISA). The mRNA and the protein of AdipoRs in the retina were detected with qRT-PCR and western blotting, respectively. The unpaired Student *t* test was used to assess the significance between the T1DM and the control groups, with p<0.05 regarded as statistically significant.

**Results:**

APN, AdipoR1, and AdipoR2 were identified in the neural retina and in the RPE-choroid of humans and mice. AdipoR1 was found in the internal limiting membrane and in the outer segments of the photoreceptors in human and mouse retinas, whereas no noticeable AdipoR2 expression was seen in the retinal frozen sections of human and mouse eyes. Compared to the control group, APN and AdipoR1 expression in the retina was elevated in the T1DM group, but AdipoR2 expression remained unchanged.

**Conclusions:**

We demonstrated that APN, AdipoR1, and AdipoR2 exist in human and mouse retinas and that retinal APN and AdipoR1 protein levels are elevated in T1DM mice, implying that the APN-AdipoR1 axis may be activated in the diabetic retina. In contrast, AdipoR2 appears to play a minor role in this pathological process.

## Introduction

Adiponectin (APN, Acrp30, or CBP28) is secreted by adipose cells and mimics many metabolic actions of insulin [1]. APN shares sequence homology with a family of proteins showing a modular design containing a C-terminal complement factor C1q-like globular domain, and the C-terminal globular domain of APN is also strikingly similar to that of tumor necrosis factor-alpha [1]. APN is involved in a wide variety of physiologic processes, including energy metabolism, inflammation, and vascular physiology, via actions on a broad spectrum of target organs, such as the liver, skeletal muscle, and vascular endothelium [2]. In addition to possessing insulin-sensitizing and anti-inﬂammatory properties, APN also exerts a pivotal role in vascular protection through the activation of multiple intracellular signaling cascades [2]. APN exerts its physiologic effects predominantly via the APN receptors, AdipoR1 and AdipoR2. These contain seven trans-membrane domains but are structurally and functionally different from G protein-coupled receptors [3].

The biologic effects of APN are complex, and the mechanisms by which APN acts are poorly understood [4]. Clinical studies regarding the relationship between the plasma adiponectin level and diabetic retinopathy (DR) have been inconclusive [5,6]. Recently, we found that the concentration of APN in the aqueous humor was higher in patients with DR [7], implying that APN may be associated with this condition. However, to our knowledge, no studies have investigated whether APN and its receptors are located in the retina. Here, we examined the messenger RNA (mRNA) and protein expression of APN and its receptors (AdipoRs) in human and mouse retinas and in the retinal pigment epithelium (RPE)-choroid complex. In addition, we investigated whether APN and AdipoRs were associated with diabetes.

## Methods

### Human materials and animals

Thirty eyecup specimens from 20 donors were obtained from the Chongqing Eye Bank. The average age of the 12 male and eight female donors was 37±14.4 years old. The deceased did not have any eye-related disease before death. The eyeball specimens were removed within 1 h of death and then stored at −20 °C. The eyes were dissected within 30 min after the corneas were removed. *C57BL/6J* and eNOS-knockout (*eNOS*^−/−^) mice were purchased from the Jackson Laboratory (Bar Harbor, ME) and bred in the animal facilities of Chongqing Medical University. All animal procedures performed in this study complied with the ARVO Statement for the Use of Animals in Ophthalmic and Vision Research and were approved by the Animal Care and Use Committee of the First Affiliated Hospital of Chongqing Medical University (ID#2011–22).

### Induction of diabetes model and detection of blood glucose level

Male *eNOS*^−/−^ mice aged 6–8 weeks received an intraperitoneal injection of 60 mg/kg streptozotocin (STZ; Sigma-Aldrich, St. Louis, MO) dissolved in 0.01 M sodium citrate buffer (pH 4.5) on three successive days, using a procedure described previously [8]. Blood glucose was measured 1 week after the injection with STZ and again 1 and 2 months later. The whole blood from the tail vein of the mice was used. The blood glucose level was detected with a quick blood glucose monitor (Leapon, Beijing, China). Mice with fasting blood glucose levels higher than 13.9 mM in the three measurements after the STZ injection were defined as diabetic. Age-matched, nondiabetic *eNOS*^−/−^ and *C57BL/6* mice were used as the control.

### Immunofluorescence

Immunofluorescence was performed using previously described protocols [9,10]. Briefly, the mouse eyes and the human eyeballs were immersed in 4% (wt/vol) paraformaldehyde for 3 h. The tissues were embedded in optimum cutting temperature compound in liquid nitrogen. Frozen sections 10 μm thick were cut through the cornea-optic nerve axis and mounted on polylysine-coated slides. The sections were immersed with 5% donkey serum in PBS (140 mM NaCl, 2.7 mM KCl, 10 mM Na_2_HPO_4_, 2 mM KH_2_PO_4_) for 30 min and incubated overnight at 4 °C with rabbit anti-AdipoR1 antibody (1:100; Santa Cruz Biotechnology, Santa Cruz, CA) and goat anti-AdipoR2 antibody (1:100; Santa Cruz Biotechnology). Some sections were incubated overnight at 4 °C with PBS as a negative control group. The sections were washed and then incubated with secondary antibody for 45 min. Images were captured with a fluorescence microscope (Leica, Bannockburn, IL).

### Enzyme-linked immunosorbent assay

The concentration of the APN protein was measured with enzyme-linked immunosorbent assay (ELISA) kits for mouse and human (R&D system, Minneapolis, MN) [7,11]. The retina and the RPE-choroid complex were dissected and then homogenized and solubilized in ice-cold PBS containing protease inhibitors. A multifunction microplate reader (Molecular Devices, Sunnyvale, CA) was used to measure the concentration of the APN protein. Each sample was measured three times, and the averaged reading was recorded. The data are shown as mean±standard deviation (SD).

### Quantitative real-time polymerase chain reaction

The retinal and the RPE-choroid complex tissues were harvested from the human and mouse eyeballs separately. Total cellular RNA was extracted with RNAiso Plus Kits (Takara Biotechnology, Dalian, China). cDNA was generated using a PrimeScript RT reagent kit (Takara Biotechnology, Dalian, China) according to the manufacturer’s instructions. The primers used were specific for human AdipoR1: Primer ID (Hs-QRP-3545237319), human AdipoR2: Primer ID (Hs-QRP-37319), mouse AdipoR1: Primer ID (MQp042945), mouse AdipoR2:Primer ID (MQp035557), and glyceraldehyde-3-phosphate dehydrogenase (glyceraldehyde 3-phosphate dehydrogenase; forward, 5′-ATG GTG AAG GTC GGT GTG AAC-3′; reverse, 5′-TTA CTC CTT GGA AG-3′; GeneCopoeia, Rockville, MD). The specificity of all the primers was verified by GeneCopoeia Inc. Quantitative real-time polymerase chain reaction (qRT-PCR) was performed in a volume of 20 µl, using an all-in-one qPCR Mix (GeneCopoeia) on a Bio-Rad c-1000 PCR machine (Bio-Rad Laboratories, Hercules, CA). The conditions were 95 °C for 10 min, followed by 40 cycles of 10 s at 95 °C, 20 s at 60 °C, and 15 s at 72 °C. Fluorescence data were acquired at 72–95 °C to decrease the amount of nonspecific signal, and amplification of specific transcripts was confirmed with melting curve profiles at the end of each PCR. The AdipoR1/R2 measurements were masked to group assignment. For each sample, qRT-PCR was performed in triplicate, and relative quantities were calculated using ABI SDS 2.0 RQ software and the 2^-ΔΔCt^ analysis method, with glyceraldehyde 3-phosphate dehydrogenase as the endogenous control [12].

### Western blotting

The retinal and the RPE-choroidal tissues harvested from the T1DM *eNOS*^−/−^ mice and the nondiabetic *eNOS*^−/−^ controls were homogenized and solubilized in ice-cold PBS containing protease inhibitors and detergent NP-40. The total protein concentration was determined with a bicinchoninic acid (BCA) protein assay kit (Beyotime Institute of Biotechnology, Haimen, China). Electrophoresis was performed on a 7.5% sodium dodecyl sulfate–polyacrylamide gel electrophoresis slab gel (Goodbio Technology, Wuhan, China), and the separated proteins were transferred to polyvinylidene difluoride membranes (Millipore, Billerica, MA). The membranes were blocked in 5% nonfat dry milk in Tris-buffered saline Tween-20 solution (TBST) for 60 min at room temperature (22–25 °C; RT). Anti-AdipoR1 (C-12) and anti-AdipoR2 (H-40; Santa Cruz Biotechnology) were used as the primary antibodies, and the membranes were incubated with the primary antibody (diluted 1:5,000) overnight at 4 °C. As a loading control, the β-actin protein was used. The designated membranes were incubated using a primary anti-β-actin antibody diluted 1:5,000 (v:v) in 3% bovine serum albumin in TBST at 4 °C overnight. The membrane was incubated with horseradish peroxidase conjugated secondary antibody for 1 h at room temperature (Kirkegaard & Perry Laboratories, Gaithersburg, MD). Following secondary antibody incubation, all the membranes were washed for 30 min, incubated for 5 min with an enhanced chemiluminescence kit (Amersham, Piscataway, NJ), and exposed to Hyperfilm enhanced chemiluminescence film (Amersham, Pittsburgh, PA). Densitometric analysis was performed with Image J software (Version1.43, Broken Symmetry Software, Bethesda, MD). For each experiment, the measurements were repeated three times.

### Statistical analysis

Statistical analyses were undertaken using SPSS (Version 17.0, SPSS, Chicago, IL). The unpaired Student *t* test was used to assess the significance between the two groups. p<0.05 was regarded as statistically significant.

## Results

### Blood glucose levels of type 1 diabetes mellitus and age-matched control mice

The T1DM group had higher blood glucose compared with the nondiabetic *eNOS*^−/−^ mice and the nondiabetic C57 mice at different time intervals after the intraperitoneal injection of STZ (p<0.001). The results confirmed that T1DM was successfully induced with STZ in the treated mice (Table 1). Data were shown as mean±SD (n=30/group).

**Table 1:** Mouse blood glucose levels at 1 week,1 month and 2 months after STZ injection

	1 week	1 month	2 months
T1DM (mmol/l)	16.4±1.39***	24.9±1.71***	29.7±2.00***
NDM *eNOS*^−/−^ (mmol/l)	6.53±0.75	6.58±0.92	6.47±1.41
NDM *C57BL/6* (mmol/l)	6.39±0.38	6.36±1.15	7.18±1.09

Data are expressed as the mean±SD. The significantly different from age-matched control mice (***p<0.001). (NDM *eNOS*^−/−^: non-diabetic *eNOS*^−/−^ mouse; NDM *C57BL/6:* non-diabetic *C57BL/6 mouse.* (n=30/group).

### Expression of adiponectin and adiponectin receptors in humans

**Figure 1:**
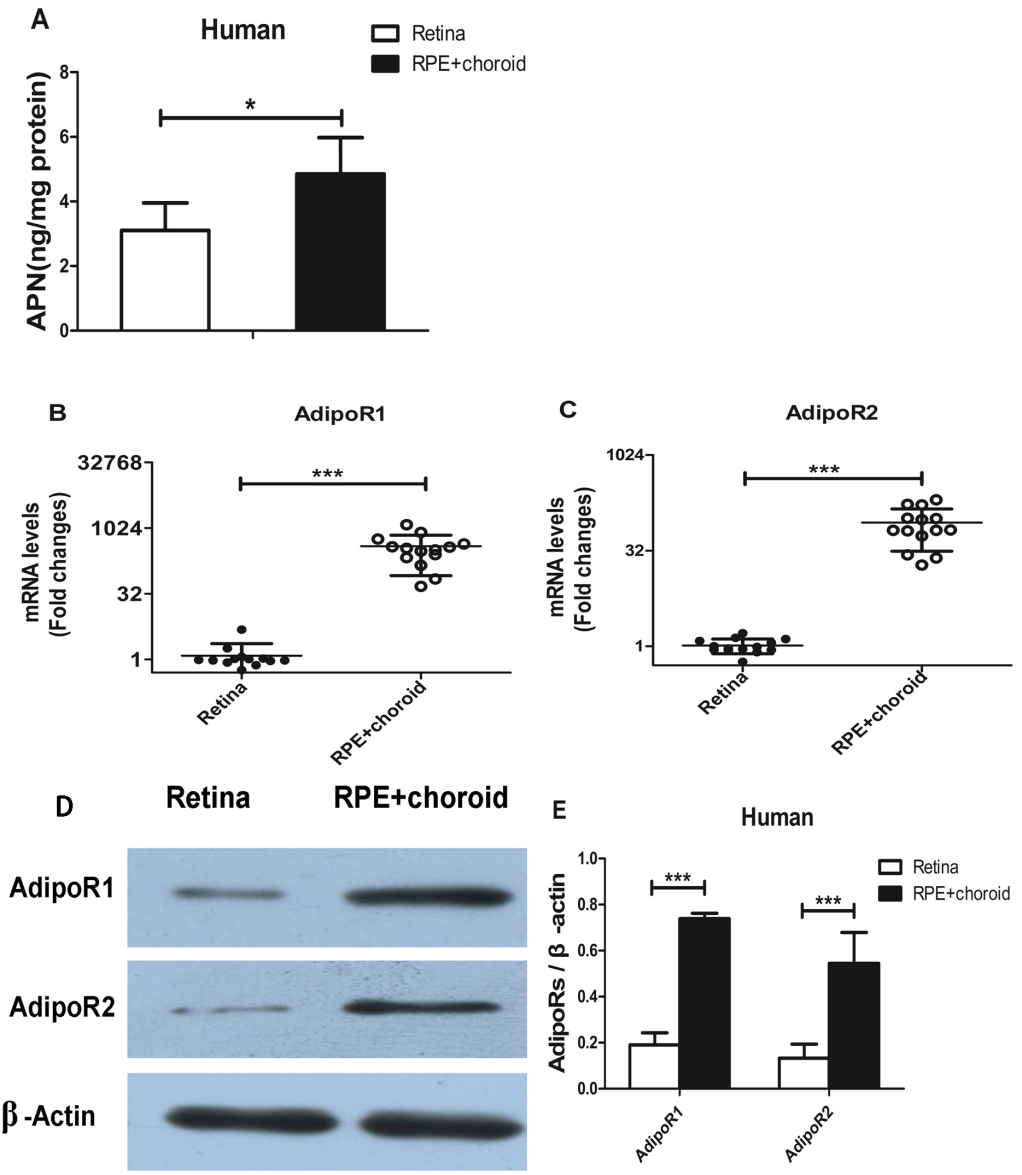
Adiponectin and its receptors expression in the retina and the retinal pigment epithelium-choroid in humans. **A**: Picture represented Adiponectin (APN) protein levels in the retina and the retinal pigment epithelium (RPE)-choroid in human eyeballs. The protein levels of APN in the retina were lower than in the RPE-choroid (n=7, *p<0.05). **B**, **C**: Picture represented Adiponectin receptors (AdipoR1 and AdipoR2) messenger RNA (mRNA) expression in the retina and the RPE-choroid in human. The adiponectin receptors (AdipoRs) mRNA levels in the retina were much lower than those in the RPE-choroid (n=14, ***p<0.001). **D**: Picture represented the AdipoRs protein levels in the retina and the RPE-choroid in human as determined with western blotting. **E**: Picture represented densitometry analysis of the AdipoRs protein levels in the retina and the RPE-choroid in human. AdipoR1, and AdipoR2 protein expression in the RPE-choroid were higher than in the retina (n=6, *p<0.05).

In the human samples, the enzyme-linked immunosorbent assay results indicated that the APN concentrations were 3.10±0.30 ng/mg protein in the retina and 4.85±0.39 ng/mg protein in the RPE-choroid (p<0.05; Figure 1A; n=7). AdipoR1 and AdipoR2 mRNA expression was detected with real-time PCR in the RPE-choroid and in the retina of the human eye. The AdipoRs mRNA levels in the retina were much lower than those in the RPE-choroid (p<0.001; Figure 1B,C; n=14) Consistent with the AdipoRs mRNA, western blotting showed that the protein expression of AdipoR1 and AdipoR2 in the RPE-choroid was higher than in the retina (Figure 1D,E; n=6).

### Expression of adiponectin and adiponectin receptors in mice

**Figure 2:**
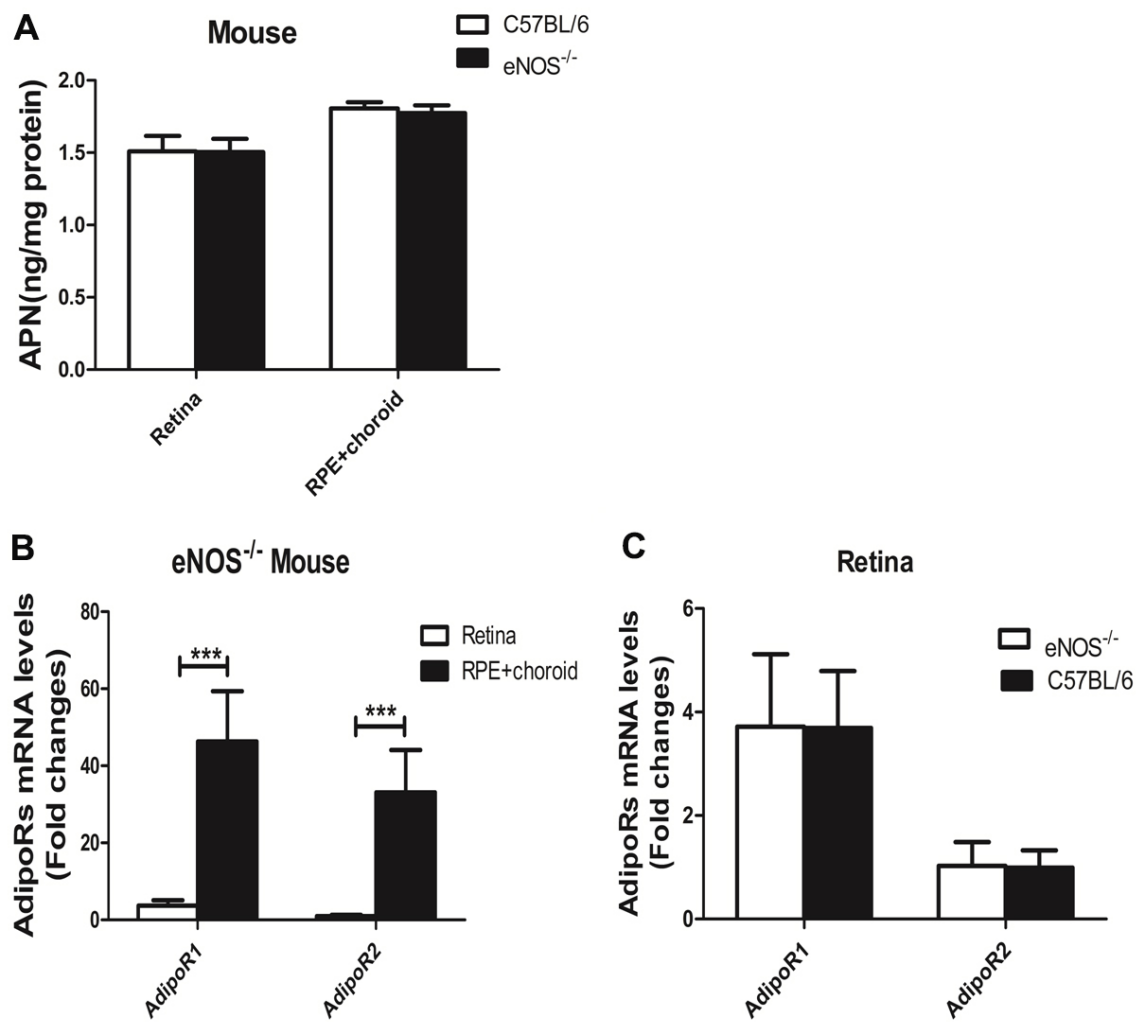
Adiponectin and adiponectin receptors expression in the retina and the retinal pigment epithelium-choroid. **A**: Image showed APN protein levels in the retina and in the RPE-choroid of the *eNOS^−/−^* and C57BL/6 mice. The protein levels of APN in the retina were lower than in the RPE-choroid (n=10,*p<0.05), and there was no significant difference in either the retina or the RPE-choroid between the *eNOS^−/−^* mice and the C57BL/6 mice. **B**: Image showed AdipoRs mRNA expression in the retina and the RPE-choroid of the *eNOS^−/−^*mice. The mRNA levels of AdipoR1 and AdipoR2 in the RPE-choroid were significantly higher than in the retina (n=11, ***p<0.001). **C**: Image showed retinal AdipoRs mRNA levels in *eNOS^−/−^* and *C57BL/6* mice. The AdipoRs mRNA levels in the retina did not show a significant difference in the *eNOS^−/−^* mice compared with the *C57BL/6* mice. Data were shown as mean±SD.

In the *eNOS*^−/−^ mice, the APN protein concentrations were 1.46±0.01 ng/mg in the retina and 1.69±0.04 ng/mg in the RPE-choroid. In the *C57BL/6* mice, the APN concentrations were 1.51±0.06 ng/mg in the retina and 1.80±0.02 ng/mg in the RPE-choroid. In the *eNOS*^−/−^ and *C57BL/6* mice, the APN protein levels in the retina were lower than those in the RPE-choroid. However, there was no significant difference in the expression levels in the retina or the RPE-choroid of the *eNOS*^−/−^ and *C57BL/6* mice (Figure 2A; n=10). For the *eNOS*^−/−^ mice, the mRNA levels of AdipoR1 and AdipoR2 were significantly higher in the RPE-choroid than in the retina (p<0.001). Compared to the retina, the AdipoR1 mRNA level was 12-fold higher and the AdipoR2 mRNA level was 33-fold higher in the RPE-choroid (Figure 2B; n=11). The AdipoRs mRNA levels in the retina did not show a significant difference in the *eNOS*^−/−^ mice compared with the *C57BL/6* mice (Figure 2C).

### Immunofluorescence of adiponectin receptors in human and mouse eyes

**Figure 3:**
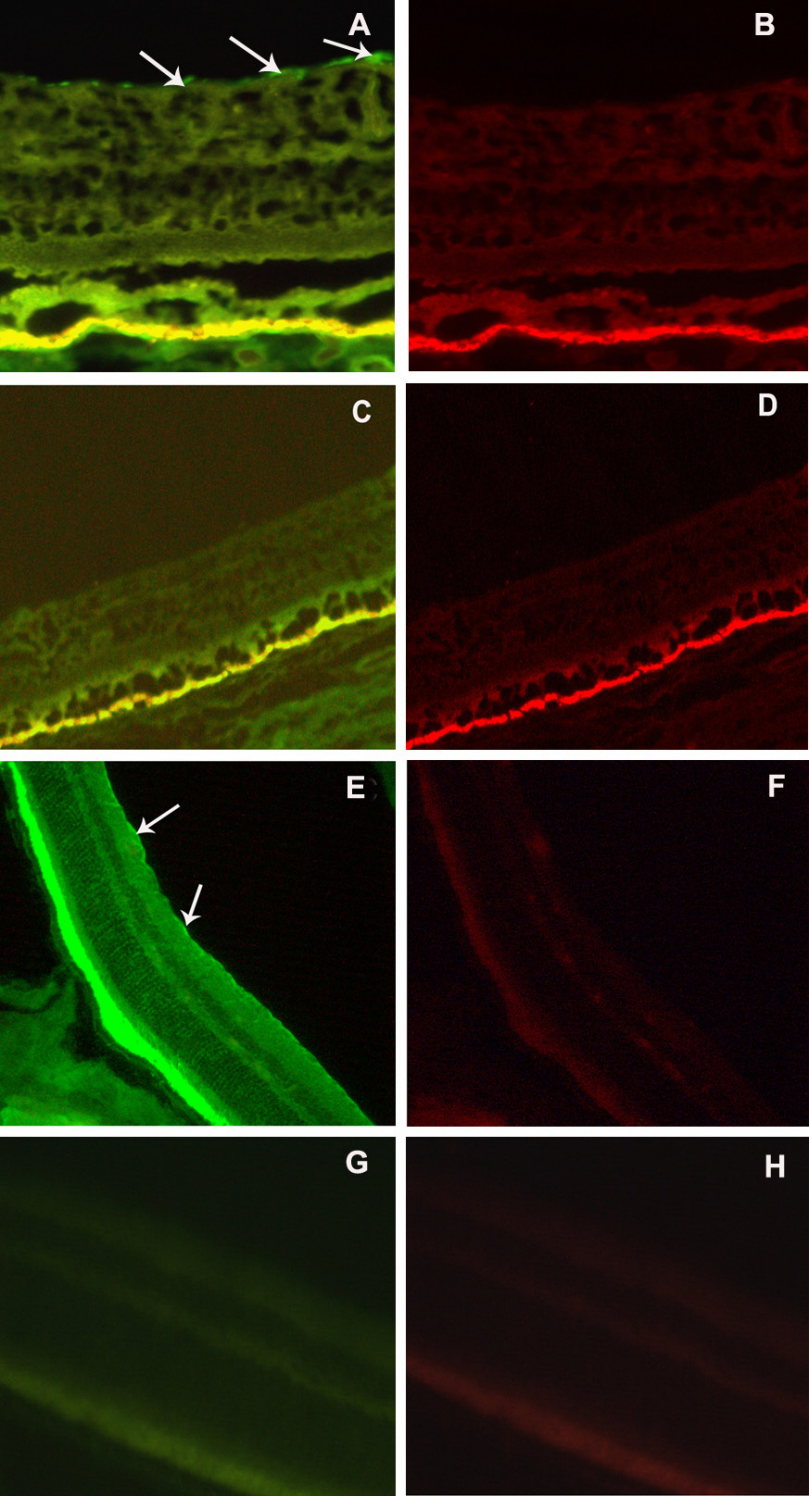
Immunofluorescence of adiponectin receptors in the human and mouse radial sections. **A**: Adiponectin receptor 1 (AdipoR1) was mainly localized at the internal limiting membrane (ILM) and the retinal pigment epithelium of the retina in humans. **B**: AdipoR2 expression was not observed in the sections of the human eyeball. **C**, **D**: Specific staining was not observed in the sections of the human eyeball in the negative control group. **E**: AdipoR1 was located in the photoreceptor outer segments and in the internal limiting membrane of the retina in the *eNOS*^−/−^ mice. **F**: AdipoR2 was not clearly detected with the fluorescence microscope in the retina of the *eNOS*^−/−^ mice. **G**, **H**: Specific staining was not observed in the sections of the *eNOS*^−/−^ mice eyeball in the negative control group. Arrows indicate AdipoR1 in the ILM layer. (The calibration bar were 25 μm for **A**–**B**, and 50 μm for **C**–**D**. The images were obtained at 400X magnification for **A**–**D** and **G**–**H**, and 200X magnification for **C**–**F**).

Immunofluorescence indicated that AdipoR1 was localized at the internal limiting membrane layer and the outer segments of the photoreceptors of the human retina (Figure 3A). AdipoRs expression was not observed in the sections of human eyeballs in the negative control group (Figure 3C,D). AdipoR1 expression in the retinas of the *eNOS*^−/−^ mice was consistent with that in the human eye (Figure 3E). Although the retinal pigment epithelium layer was positive in the human and mouse eyes, we could not exclude the possibility of nonspecific staining. AdipoR2 expression was not observed in the sections of the human and mouse eyeballs (Figure 3B,F). AdipoRs expression was also not observed in the mouse eyeball sections in the negative control group (Figure 3G,F).

### Expression of adiponectin and adiponectin receptors in the retinas of mice with type 1 diabetes mellitus

**Figure 4:**
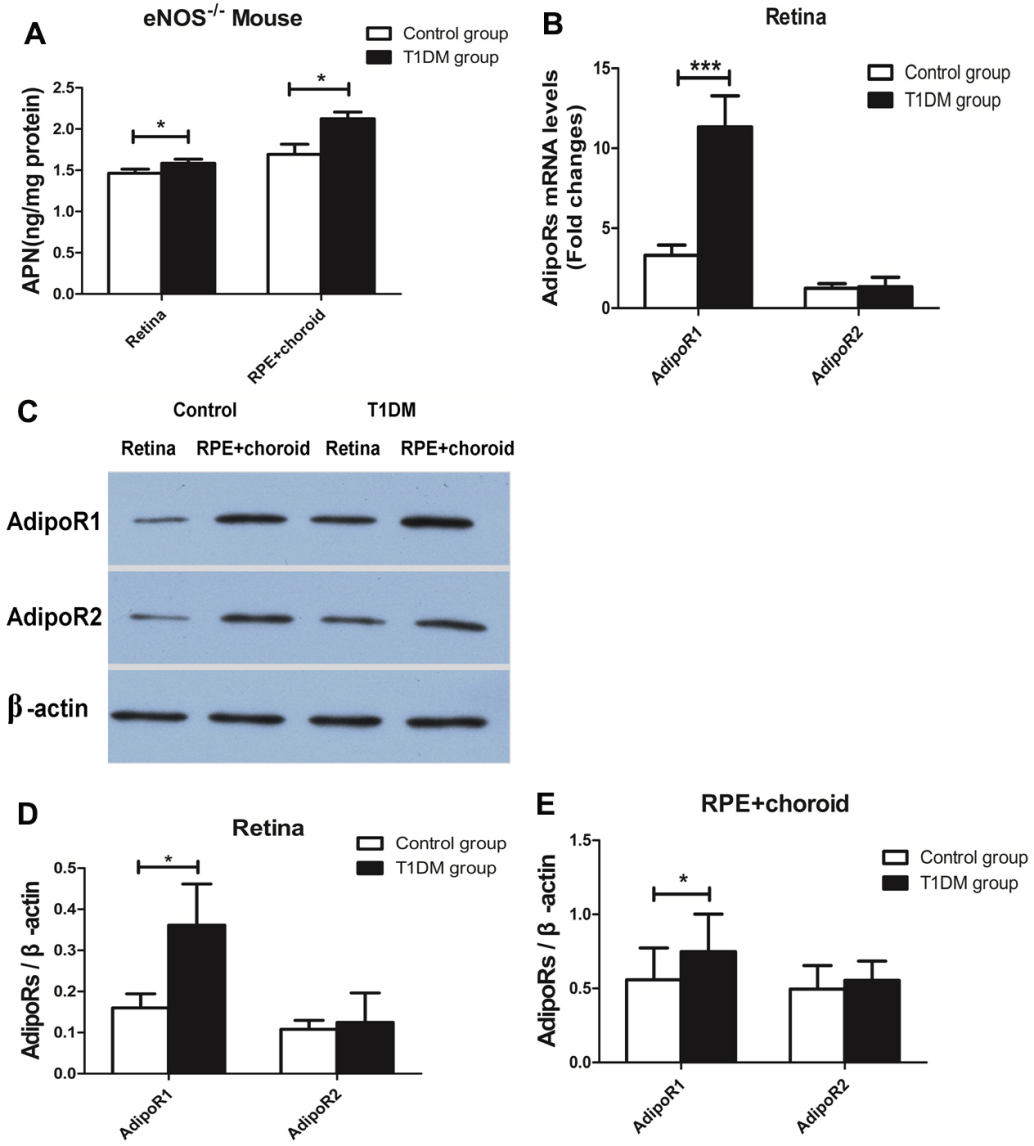
The expression of adiponectin and adiponectin receptors in the retina and the retinal pigment epithelium-choroid in mice with type 1 diabetes mellitus. **A**: Picture illuminated APN protein levels in the retina and the RPE-choroid of the type 1 diabetes mellitus (T1DM) group and the control group. The APN protein levels in the retina and the RPE-choroid of the T1DM group were higher than those in the control group (n=8, *p<0.05). **B**: Picture illuminated the AdipoRs mRNA levels in the retina of the *eNOS^−/−^* mice control group and the *eNOS^−/−^* mice T1DM group. The AdipoR1 mRNA levels in the retina were significantly increased in the T1DM group compared to the control group (n=11, ***p<0.001), but there was no obvious difference in the AdipoR2 mRNA levels between the T1DM group and the control group. **C**: Picture illuminated the AdipoRs protein levels in the retina and the RPE-choroid in the control group mice and in the T1DM group as determined with western blotting. **D**, **E**: Densitometry analysis showed that the AdipoRs protein was expressed in the control group and in the T1DM group. The AdipoR1 protein levels were higher in the T1DM group than in the control group in the retina and the RPE-choroid, and there was no difference in the AdipoR2 protein in the retina and the RPE-choroid between the T1DM group and the control group (n=6, *p<0.05). Data were shown as mean±SD.

In the T1DM group, the concentrations of the APN protein were 1.58±0.01 ng/mg in the retina and 2.13±0.03 ng/mg in the RPE-choroid, whereas in the control group, the concentrations were 1.46±0.01 ng/mg in the retina and 1.69±0.04 ng/mg in the RPE-choroid. There was a significant difference between the two groups in the APN protein level of the retina and the RPE-choroid *(*p*<*0.05; Figure 4A; n=8). The AdipoR1 mRNA levels in the retina were significantly increased in the T1DM group compared to the control group (p<0.001).There was no obvious difference in the AdipoR2 mRNA levels between the T1DM group and the control group (Figure 4B; n=11). The AdipoR1 protein levels in the retina and the RPE-choroid were higher in the T1DM group than in the control group (p<0.05, Figure 4C,D; n=6). Consistent with the data from the qPCR results, there was no difference in AdipoR2 protein expression in the retina and the RPE-choroid between the T1DM group and the control group (p>0.05, Figure 4C,E).

## Discussion

APN, produced mainly by adipose tissue, performs numerous biologic actions. APN suppresses the production of inflammatory factors and the formation of neovascularization; APN also reduces pericytes and the apoptosis of endothelial cells [13,14]. Emerging evidence suggests that APN may be associated with several retinal diseases [1,4,5,15]. The overexpression of APN attenuated laser-induced choroidal neovascularization, a model of the wet form of age-related macular degeneration [4]. Due to APN’s association with enhanced insulin sensitivity, APN deficiency is believed to be closely related with the pathological progression of diabetic retinopathy [4,6,16-18]. To further understand the underlying mechanisms of APN in the pathogenesis of DR, determining the distribution of APN and its receptors in normal and diseased retinas is crucial. However, to our knowledge, such data are still not available in the literature.

### Expression of adiponectin and its receptors in the retina

In this study, we have shown that APN and one of its receptors, AdipoR1, are present in the retinas of humans and mice. As we used the RPE-choroid complex in this study, we could not detect the distribution of AdipoRs and APN in the choroid. However, it has been reported that APN and AdipoR1 are present in the endothelium of newly formed vessels of the mouse choroid [15]. As adipose tissues are the main source of APN production, we postulate that APN in the retina is derived from circulating blood. The fact that the concentration of APN in the choroid is higher than that in the retina and that the concentration of APN in plasma is much higher than that in the aqueous humor support this notion [19,20]. Three isoforms of APN, a low-molecular weight (LMW) trimer, a middle-molecular weight (MMW) hexamer, and a multimeric high-molecular weight complex, have been identified. Their molecular weight are higher than the molecular weight limits of the inner and outer blood–retinal barrier (BRB) [21]. In this regard, we postulate that there are transport systems for APN between the systemic circulation and the retina. Previous studies suggested that APN might pass through the blood–brain barrier using a transport mechanism mediated by receptors, similar to the mechanism by which leptin permeates from the blood to the cerebrospinal fluid [20,22-24]. However, whether and what kind of AdiopRs are involved in transporting APN into the retina in the eye remains unclear.

APN is involved in various biologic processes mediated via its receptors. Two types of receptors have been documented. AdipoR1 is more prominent in adenine mononucleotide protein (AMP)-activated protein kinase (AMPK) phosphrylation, and AdipoR2 is involved in peroxisome proliferator-activated receptor α activation [25]. Endothelial AMPK signaling is essential for angiogenesis under conditions of hypoxia but dispensable in normoxic cells. AMPK activation by APN-AdipoR1 can activate angiogenic cellular responses in normoxic endothelial cells [26]. AdipoR2 was associated with the activation of peroxisome proliferator-activated receptor α pathways and the inhibition of inflammation and oxidative stress, which are involved in the induction of insulin resistance [27]. In this study, we found that AdipoR1 was present in the retina and the choroid of humans and mice. In both species, AdipoR1 was expressed mainly in the internal limiting membrane layer and in the outer segments of the photoreceptors. Although we detected AdipoR2 at the protein level, we did not observe AdipoR2 expression in the retinal sections with immunofluorescence. The deviation in the expression of the two APN receptors, together with the presence of APN in the eye, implies that AdipoR1 is functional, whereas AdipoR2 plays a minor role in the retina.

### Expression of adiponectin and its receptors in the mouse model of diabetic retina

The *eNOS^−/−^* mice exhibited more profound retinal vascular lesions 2 months after the injection of STZ [8]. In this study, we used this mouse model of diabetic mellitus to investigate whether the retinal APN system was affected in this pathological condition. We found that the APN and AdipoR1 protein levels were significantly increased in the diabetic retina, whereas AdipoR2 protein expression remained unchanged. Together with our previous findings that the concentration of aqueous APN in proliferative DR patients is significantly higher than in nondiabetic subjects [7], these data suggest that the APN-AdipoR1 axis is activated in the diabetic retina. Three possibilities may explain the elevation of APN in the retina. First, the breakdown of the BRB, a hallmark of DR [28], may cause leakage of APN into the retina. Second, based upon the analysis above, as the mRNA levels of APN and AdpoR1 are highly elevated in diabetic retina, APN may be actively transported through the BRB into the retina by AdipoR1. Third, the AdpoR1 protein and at least part of the APN protein might be generated locally in the diabetic condition. Low concentrations of APN have been detected in the skeletal muscles, liver, colon, heart muscle, salivary glands, and placenta [29,30]. APN was also detected in cerebrospinal fluid and breast milk in low concentrations [31]. We presume that parts of APN and AdipoRs may be endogenous or locally expressed.

Given the fundamental roles of APN in suppressing inflammation and neovascularization, the elevation of the APN-AdipoR1 axis in the diabetic retina might represent a mechanism of compensation [19,32,33], which protects and repairs vascular endothelial damage. Emerging evidence suggests that DR is one of the most common microvascular complications of diabetes and that APN is protective against vascular dysfunction induced by diabetes mellitus through multiple favorable effects on glucose metabolism, as well as on vascular function [4,19,32,34-37]. The vasoprotective actions of APN include the attenuation of the production of reactive oxygen species in endothelial cells and the reduction of vascular smooth muscle cell proliferation and migration [38]. Additionally, APN potently inhibited vascular endothelial growth factor (VEGF)-induced ROS generation when combined with the anti-inﬂammatory actions of APN, indicating that APN has a broad antioxidant role in the vasculature [39]. APN also suppressed pathological microvessel formation in the retina through modulation of tumor necrosis factor-alpha inflammatory responses and decreased VEGF and VEGF-R2 expression [15,36].

In summary, we have demonstrated that APN, AdipoR1, and AdipoR2 exist in human and mouse retinas. The APN-AdipoR1 axis appeared to be activated in the T1DM *eNOS^−/−^* mice, whereas AdipoR2 expression was unchanged. The activation of the APN-AdipoR1 axis might play a compensatory role in T1DM. However, the exact mechanisms are still unclear, and future studies are required to discover the role of APN signaling in retinal cells.
